# Characterization of New Substrates Targeted By *Yersinia* Tyrosine Phosphatase YopH

**DOI:** 10.1371/journal.pone.0004431

**Published:** 2009-02-16

**Authors:** María Luisa de la Puerta, Antonio G. Trinidad, María del Carmen Rodríguez, Jori Bogetz, Mariano Sánchez Crespo, Tomas Mustelin, Andrés Alonso, Yolanda Bayón

**Affiliations:** 1 Instituto de Biología y Genética Molecular, CSIC-Universidad de Valladolid, Valladolid, Spain; 2 Program of Inflammation, Inflammatory and Infectious Disease Center, and Program of Signal Transduction, Burnham Institute for Medical Research, La Jolla, California, United States of America; Instituto Oswaldo Cruz and FIOCRUZ, Brazil

## Abstract

YopH is an exceptionally active tyrosine phosphatase that is essential for virulence of *Yersinia pestis*, the bacterium causing plague. YopH breaks down signal transduction mechanisms in immune cells and inhibits the immune response. Only a few substrates for YopH have been characterized so far, for instance p130Cas and Fyb, but in view of YopH potency and the great number of proteins involved in signalling pathways it is quite likely that more proteins are substrates of this phosphatase. In this respect, we show here YopH interaction with several proteins not shown before, such as Gab1, Gab2, p85, and Vav and analyse the domains of YopH involved in these interactions. Furthermore, we show that Gab1, Gab2 and Vav are not dephosphorylated by YopH, in contrast to Fyb, Lck, or p85, which are readily dephosphorylated by the phosphatase. These data suggests that YopH might exert its actions by interacting with adaptors involved in signal transduction pathways, what allows the phosphatase to reach and dephosphorylate its susbstrates.

## Introduction

*Yersinia pestis*, the bacterium responsible for plague, has caused devastating pandemics in the past [1], [2]. The bubonic plague is transmitted to humans by blood-sucking fleas infected from animal reservoirs, mostly rats and other rodents [3]. Once in the organism, *Yersinia* presents tropism for lymphoid tissue, where the bacterium proliferates rapidly in the extracellular space, avoiding the host immune system and causing an intensive lymphadenitis within 2 to 6 days [1], [2]. Another variant, the pneumonic plague, is caused by inhaled bacteria. This less usual and even more dangerous form of plague is difficult to treat and often results in death [4]. Although there are several treatments available, such as vaccines [5], [6] and antibiotics, they are not very effective, especially against pneumonic plague. Moreover, *Y. pestis* has started to be considered as a potential tool for bioterrorism due to its rapid replication and effective-immune evading ability.

*Y. pestis* contains an extracromosomal 70-kb virulence plasmid [7], [8], [9], which is essential for *Yersinia* pathogenity and encodes the Yop (*Yersinia* outer proteins) effector proteins and the proteins forming a type III secretion system. The direct injection of the effector proteins by this secretion apparatus enables the bacterium to survive and proliferate in the lymphoid tissues [10], [11]. An essential virulence factor of *Yersinia* is YopH, a 51-kd protein tyrosine phosphatase (PTP) [12], [13] with a C-terminal catalytic domain that shares structural similarities to that of eukaryotic PTPs [14], followed by a Pro-rich sequence and a multifunctional N-terminal domain, which binds tyrosine phosphorylated target proteins [15], [16]. Bacterial injection of YopH into phagocytic cell types causes the inhibition of the inflammatory response of the host to the bacteria by processes such us disruption of focal adhesions [17], [18] and inhibition of phagocytosis [19], [20], tumor necrosis factor α release, and oxidative burst [21], [22]. YopH also impairs T and B lymphocyte function [23] at very early stages preventing a successful adaptive immune response which is crucial for the survival of the bacteria in the lymph nodes of the infected host. Several proteins have been identified as YopH substrates in different cell types. In epithelial cells, the adaptors p130Cas (p130Crk-associated substrate) and paxilin, and the tyrosine kinase FAK (focal adhesion kinase). In macrophages, p130Cas, Fyb (Fyn binding protein) [24], SKAP-HOM (SKAP55 homologue) [25], and Pyk, a tyroine kinase homologous of FAK. And in T-cells, Lck, LAT, and SLP-76 [26], [27]. The majority of these proteins fall in two classes: tyrosine kinases and adaptors. Notably, these proteins participate in pathways involved in phagocytosis and activation of signal transduction in the early stages of the immune response in haematopoietic cells.

Given the complex nature of the signalling pathways activated in the immune responses and the numerous proteins involved, we hypothesized that to inhibit the immune response with such potency, YopH should have a wide specificity so it could target a broad range of proteins. As a first approach to identify new YopH substrates, we planned biochemical experiments to demonstrate these interactions. Our results showed that YopH binds p85, Gab1, Gab2, and Vav, although, YopH only dephosphorylated p85. In this sense, we proposed that binding to the adaptors Gab1, Gab2 and Vav could localize YopH at sites where signalling complexes are formed. By targeting these complexes, YopH impairs the adequate immune response by the host. The findings here described will help understand the molecular mechanisms dependent on YopH that are used by *Yersinia pestis* to evade the immune system.

## Results and Discussion

### YopH interacts with several proteins involved in signalling pathways

**Figure 1:**
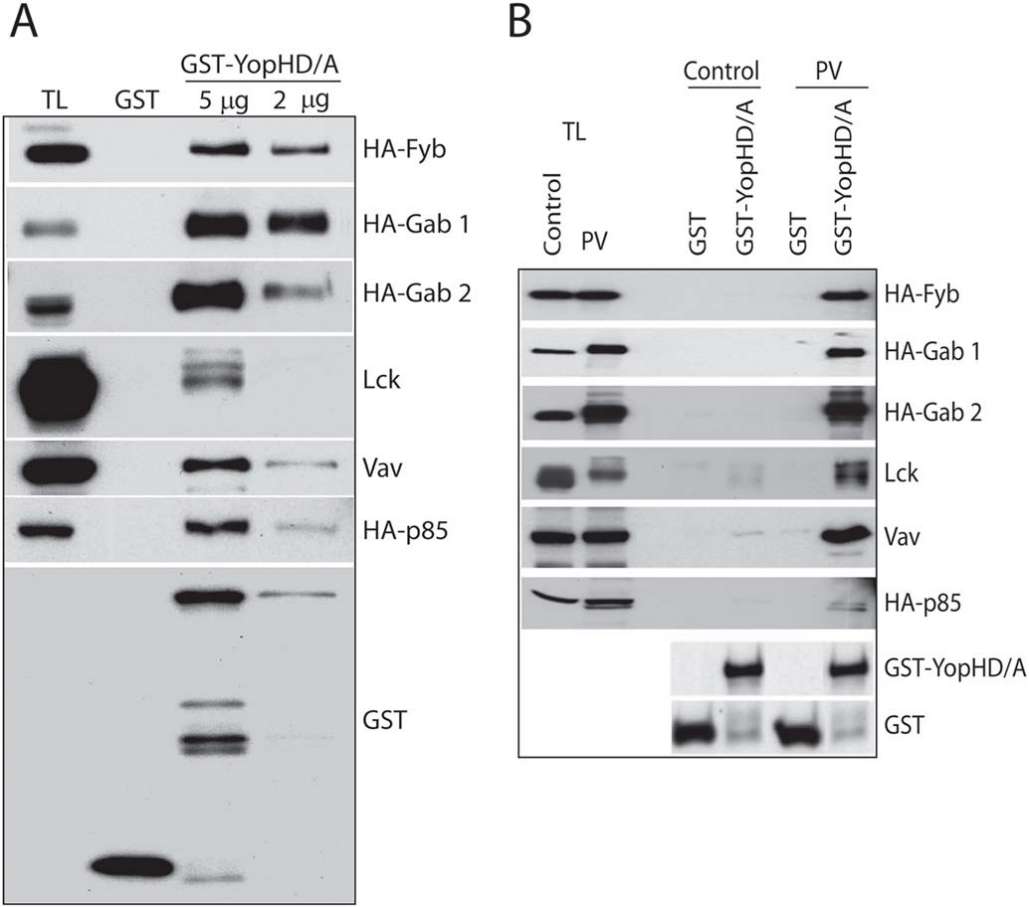
Pull-down assays with GST-YopH D356A. A, HEK293 cells expressing different proteins, either untreated (control) or treated with pervanadate (PV) to induce tyrosine phosphorylation of the proteins expressed, were lysed and probed for interaction with GST-YopH D346A or GST (5 µg each) as a negative control in pull-down assays. The specific interaction of those proteins with GST-YopH D356A was detected by Western blot with specific antibodies for Lck or Vav, and with anti-HA antibody for other proteins. B, As in A, HEK293 cells, expressing the same proteins and treated with PV, were lysed and probed for interaction with two different amounts of GST-YopH D346A (5 and 2 µg) or GST (5 µg each) as a negative control in pull-down assays. GST and GST-YopH D346A fusion protein used in these assays are shown at the lower panel from one representative blot. TL denotes total lysates of the transfected cells and corresponds to a 10% of the amount used for each pull-down assay. Assays were done independently for each protein.

YopH blocks the host immune response by targeting several signalling pathways involved in activation of immune cells. This highly active bacterial PTP inhibits phagocytosis, oxidative burst associated with this process in macrophages and neutrophils, Ca^2+^ signalling in neutrophils, and antigen induced activation of lymphocytes[28]. Integrin signalling initiated by binding of Yersinia invasion to β_1_-integrin in the host cells, as well as antigens through TCR (T-cell receptor) in lymphocytes, depend on the activation of tyrosine kinases that phosphorylate a great number of substrates involved in those pathways. Given the potency of YopH to shut down these signalling pathways, we considered that YopH could target additional proteins not identified as yet. Having this in mind, we check by biochemical methods the interaction of YopH with several signalling proteins known to be expressed in hematopioetic cells. Thus, we expressed several proteins in HEK293 cells and treated them with pervanadate to induce their tyrosine phosphorylation. Lysates were used in pull-down assays with 2 or 5 µg of a GST fusion protein of YopH substrate trapping mutant, GST-YopH D356A. We assayed several proteins for interaction with YopH, mainly tyrosine kinases like Lck, Fyn, Csk, Zap-70, Syk and the regulatory subunit of the PI3K, p85; and adaptors such as Gab1, Gab2, Cbl, Fyb, Vav, and Grb2. We used Fyb and Lck as positive controls in these assays, because they have been shown before to bind YopH [24], [26]. In our assays, GST-YopH D356A bound Fyb, Gab 1, Gab 2, Lck, Vav and p85 (Fig. 1 A), while it did not bind the rest of the proteins tested (data not shown). Some of those proteins, LAT and Zap-70, have been shown to interact with YopH but we have not been able to observe this association. Differences between our data and those of Gerke et *al.* [27] could be explain by the different technical approach used, pull-down versus immuniprecipitation of *Yersinia* infected T-cells. An alternative explanation is also possible; the presence of those proteins in the precipitates could be due to indirect interaction with other proteins present in T-cells.

Although binding of these proteins to YopH was expected to be mediated by tyrosine phosphorylation, we confirmed this finding with another experiment in which the pull-down assay was carried out with lysates from transfected HEK293 cells treated and left untreated with pervanadate. As hown in Fig. 1.B, interaction between YopH DA (D356A) is mainly detected when proteins are phosphorylated on tyrosine. Only a slight interaction is observed in the case of two proteins, Lck and Vav, in absence of pervanadate, which it is probably due to the presence of some phosphorylated tyrosine in the resting state, as it is the case of Lck Y505.

### YopH interacting proteins are not only substrates but also adaptor proteins

**Figure 2:**
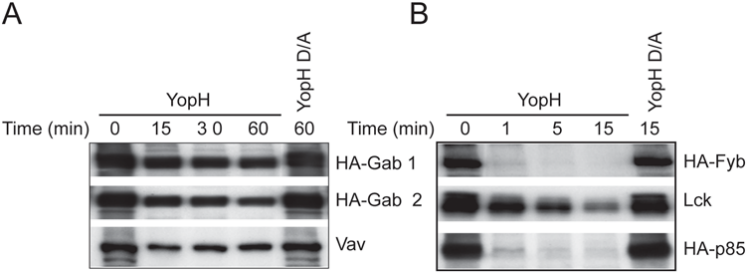
YopH dephosphorylation assay of several proteins. A, Dephosphorylation assay for HA-Gab1, HA-Gab2 and Vav at different time-points, using 1 µg of GST-YopH or GST-YopH D356A. The assay was stopped by addition of sample buffer, and after SDS-PAGE, samples were transferred to nitrocellulose and tyrosine phosphorylation was detected by Western blot with anti-phosphotyrosine antibody. B, Dephosphorylation assay for HA-Fyb, Lck and HA-p85 was carried out as in A, but using shorter incubation times. Proteins used as substrates in these assays were obtained from HEK293 transfected with the corresponding plasmids and treated with pervanadate. Proteins were immunoprecipitated and distributed equally in different tubes for the several time-points of the assay.

To demonstrate that the proteins that bound YopH were substrates of this phosphatase we carried out dephosphorylation assays *in vitro* with recombinant GST-YopH produced in bacteria using as control the inactive substrate trapping mutant GST-YopH D356A. Contrary to our expectations, Gab1, Gab2 and Vav were not dephosphorylated, even with incubations as long as 1 hour (Fig. 2A). On the other hand, as it has been shown before, Lck and Fyb were dephosphorylated [24], [26], although in the case of Lck, with a lower efficiency. In this assay, we also detected p85 dephosphorylation by YopH, with almost the same efficiency than Fyb. These data support the notion that YopH shows selectivity for some proteins as demonstrated by other researchers [19], [29]. Furthermore, we show that YopH is able to bind to some adaptor proteins without dephosphorylating them, thus YopH could associate with immune signalling complexes and, in this way, be localized to the proximity of its substrates to dephosphorylate them.

### Different YopH domains are involved in the interaction with the target proteins

**Figure 3:**
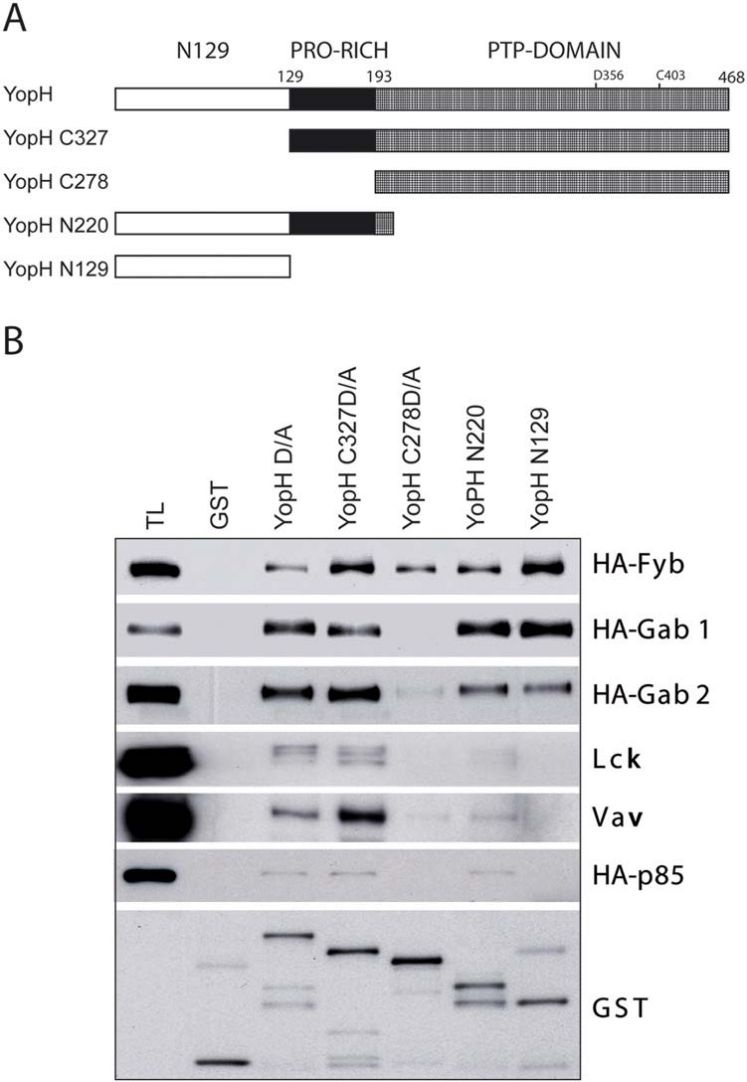
Pull-down assays with different deletion mutants of YopH. A, Schematic diagram showing the different deletion mutants of YopH used in this study. B, HEK293 cells expressing different proteins and treated with pervanadate (PV) to induce tyrosine phosphorylation of the proteins expressed were lysed and probed for interaction with GST-YopH D/A (mutation D346A), the different deletion mutants shown in A fused to GST, and GST (5 µg each) as a negative control in pull-down assays. The specific interaction of those proteins with YopH fragments was detected by Western blot with specific antibodies for Lck and Vav, and with anti-HA antibody for other proteins. An independent experiment was done for each protein. The lower panel shows a representative blot from one of the experiments to show that similar amounts of GST proteins were used in these assays.

YopH binds tyrosine phosphorylated proteins through two domains, the N-terminal domain (amino acids 1–129) and the catalytic domain (amino acids 193–468), which presents two interaction sites, the catalytic pocket and a second site on the opposite side of this domain [30]. Between these sites, there is a Pro-rich region (PRR) that may bind to SH3 domains. To analyze the interaction of YopH with the proteins studied here and based on this domain structure and on the biochemical data related to YopH substrate interaction, we generated four deletion mutants of YopH: N129 (amino acids 1–129), N220 (amino acids 1–220), C339 (amino acids 129–468), and C277 (amino acids 193–468) (Fig. 3 A). These peptides were used as GST-fusion proteins in pull-down assays using lysates from pervanadate stimulated cells. Using this approach, we observed that Fyb, a well-known YopH substrate, binds YopH through the N129 and the catalytic domain. Gab1 and Gab2 also bind through both domains but they do not bind to C227, indicating that, for binding, they required additional amino acids present in the PRR. Taking into account that Gab adaptors lack SH3 domain, association would imply another mechanism not determined yet. In the case of Vav, p85, and Lck, the stronger association was observed with the deletion mutant C339, which contains the catalytic domain and the Pro-rich region. All these proteins contain SH3 domains that might interact with the YopH PRR, thus explaining why removal of this region abrogates the interaction and why the N220 construct binds slightly to these proteins while the N129 domain shows no binding at all. According to the data shown in Fig. 3B, the proteins that bind to YopH can be divided into three groups: i) proteins that interact with both the catalytic domain and the N129 domain (Fyb), ii) proteins that interact with the extended catalytic domain, which includes PRR domain and the N129 domain (Gab1 and Gab2), and iii) proteins that only bind to the extended catalytic domain C339 (Lck, p85, and Vav).

### YopH phosphatase domain is enough for the inhibition of T cell activation

**Figure 4:**
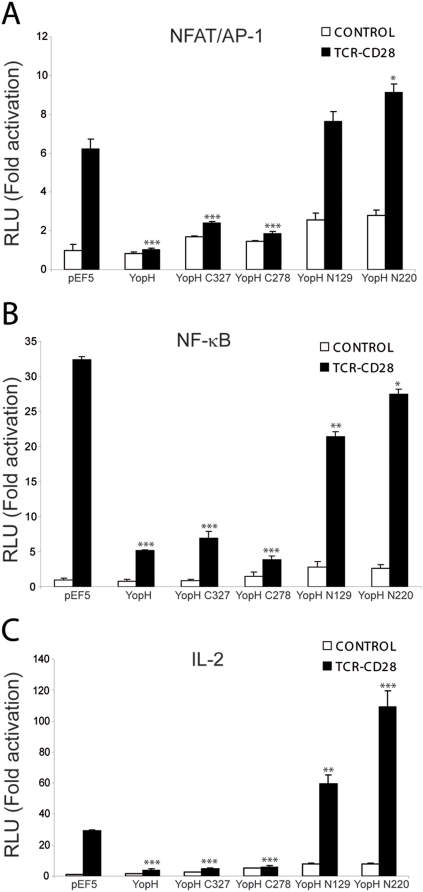
Inhibition of different luciferase transcriptional reporters by YopH deletion mutants. A, Activation of a *luciferase* reporter gene driven by NFAT/AP1. B, Activation of a NF-κB-driven *luciferase* reporter gene. C, Activation of a *luciferase* reporter gene driven by IL-2 promoter. In all cases, the data represent the mean±S.D. from triplicate determinations. *, p<0.05, **, p<0.005, ***, p<0.0005 as compared with pEF5 trasfected cells stimulated through TCR and CD28.

To determine how these deletion mutants affect signalling *in vivo*, we overexpressed them in Jurkat T-cells along with reporter plasmids that express the *luciferase* gene under the control of different promoters relevant to T-cell activation: NFAT/AP1 and NF-κB sites from the IL-2 promoter, and the minimal IL-2 promoter. In all the cases we obtained similar results (Fig. 4). N-terminal domains did not inhibit activation of these reporters and in the case of IL-2 promoter they even caused some increase over the activation produced by the stimuli alone. This increase could be due to some specific effect in which this peptide disrupts some molecular interaction of the proteins here studied or, alternatively, to an unspecific effect. On the other hand, C-terminal domains C278, which contains the PTP domain, and C339, which contains the PTP domain and the Pro-rich sequence, inhibited the activation of the reporters with a potency similar to the one exerted by the whole protein. These results show that a long exposure of cells to both YopH phosphatase and its catalytic domain inhibits activation of signal transduction pathways.

### Conclusions

In this work, we show the interaction of YopH with several proteins expressed in hematopoietic cells and involved in signalling transduction pathways, such as the adaptor proteins Gab1, Gab2; the guanine nucleotide exchange factor (GEF) for Rho-family GTPases, Vav; and p85, the regulatory subunit of the PI3Kinase. The PI3K family is involved in the innate and adaptive immune responses. Class I PI3Ks are heterodimers containing a regulatory subunit, of which p85 is the most common, and a p110 catalytic subunit. This class of PI3Kinases acts downstream of receptor tyrosine kinases activated by stimuli such as cytokines, antigen receptors (TCR, BCR, FcR), and Toll-like receptors [31], [32]. PI3K enzymes are involved in phagocytosis initiated by different pathways in immune cells [31], therefore inhibition of this pathway by YopH would inhibit uptake of *Yersinia*. Our data showing that p85 is targeted by YopH provides additional information about the mechanism by which YopH inhibits the PI3K/Akt pathway that leads to the induction of MCP-1 and IL-2 cytokines in macrophages and T cells [33], respectively. In this respect, p85 dephosphorylation by YopH would impede the recruitment of monocytes, attracted by the chemokine MCP-1, to the sites of *Yersinia* infection.

Gab1 and Gab2 belong to the Dos/Gab subfamily of scaffolds and play important roles in the function of many hematopoietic cell types. Gab1 and Gab2 are tyrosine phosphorylated upon receptor engagement by antigens and cytokines [34]. Studies on bone marrow–derived macrophages from Gab2−/− mice have shown that this protein is involved in FcγR-mediated phagocytosis. Tyrosine phosphorylation of Gab1 and Gab2 after receptor engagement allows association with p85 through its SH2 domains. Therefore, by binding to Gab adaptors, YopH might target p85 or other proteins recruited to the same signalling complex that quite likely would regulate phagocytosis in the immune system. In our hands, affinity of YopH for Gab adaptors is similar to the affinity for another well known substrate of YopH, Fyb. This data would indicate that in addition to binding directly to its substrates, YopH binds to some adaptors, what would improve its ability to gain access to substrates in signalling pathways. In other words, this strategy would allow YopH to be at the place where signalling complexes are formed to dephosphorylate its cognate substrates.

In addition to its function as GEF for Rho-GTPases that are known to regulate the actin cytoskeleton, Vav may develop adaptor like functions through the other domains present in its sequence [35]. Phosphorylation of Vav proteins has been reported in all hematopoietic cells downstream of immune receptors, including antigen receptors (TCR, BCR, FcεRI, FcγRI/II/III), integrins, cytokine receptors, and chemokine receptors. YopH does not dephosphorylate Vav, what makes it likely the use of this protein by YopH to target signalling proteins involved in activation of the cytoskeleton [36].

Our assays show that the PRR is involved in binding to most of the proteins here studied. Only Fyb was able to bind to all the YopH deletion mutants, in particular, it is the only protein that bound clearly to the catalytic domain, C278. Then, YopH is able to bind Fyb phosphorylated tyrosines either through the catalytic domain or through the N129 substrate binding domain and to the Fyb SH3 domain through the PRR. Gab2 shows a slight binding to the C278 catalytic domain while Gab1 did not bind this peptide. The fact that Gab proteins, which lack SH3 domains, bind to the C339 peptide that contains the PRR in addition to the phosphatase domain suggest that this PRR contributes notably to the interaction and this binding might be mediated by a mechanism other than the canonical association of SH3 domains with Pro motifs. The other 3 proteins here studied, Lck, Vav, and p85, bind mainly to YopH through the C339 peptide and only weakly with the N220. From our data, we can conclude than tyrosine phosphorylation is the main requirement for YopH binding and although YopH PRR contributes to this binding, its role is not clear at least in the case of Gab proteins since this region increase association to YopH and those proteins lack SH3 domains.

In summary, herein we present biochemical data supporting the interaction of the *Yersinia* phosphatase YopH with Gab1, Gab2, Vav, and the p85 regulatory subunit of PI3K. Only p85 was dephosphorylated by YopH, suggesting that the other proteins are used by YopH to target signalling complexes in the immune cells. These results, here presented, broaden the knowledge about substrate repertoire of YopH and help to understand YopH inhibitory potency on cell host signalling pathways.

## Materials and Methods

### Antibodies and reagents

Tissue culture reagents were from Cambrex (Verviers, Belgium). The 12CA5 anti-hemagglutinin (HA) monoclonal antibody (Ab) was from Roche (Indianapolis, IN USA), anti-HA clone HA.11 was from Covance (Berkely, CA USA), anti-GST (Glutathione S-transferase) was from Santa Cruz Biotechnology Inc. (Santa Cruz, CA USA), anti-β-actin mAb (monoclonal Ab) was from Sigma Chemical Co. (St. Louis, MO USA). Anti-CD3 (UCHT1) and CD28 (clone CD28.2) Ab were from BD Pharmingen (Franklin Lakes, NJ USA). The anti-phosphotyrosine mAb 4G10 was from Upstate Biotecnology, Inc. (Lake Placid, NY).

### Plasmids and mutagenesis

Plasmids encoding HA-YopH and HA-YopH D356A and Lck were described before [26], as well as p85 vector [37]. Fyb expresion plasmid was kindly provided by Christopher E. Rudd, Gab1 and Gab2 were generous gifts from Gen-Sheng Feng, and Vav expression vector was kindly provided by Xose Bustelo. Standard molecular biology techniques were used to generate the different constructions used in this study. YopH deletions were done by PCR using appropriate primers. Mutagenesis was performed using the QuickChange site-directed mutagenesis kit (Stratagene, San Diego, CA) as described by the manufacturer.

### Cell culture and transfections

Jurkat T leukemia cells were kept at logarithmic growth in RPMI 1640 medium supplemented with 10% fetal bovine serum, 2 mM L-glutamine, 1 mM sodium pyruvate, nonessential amino acids, 100 U/ml penicillin G, and 100 µg/ml streptomycin. Transfection of Jurkat T cells was performed by electroporation as described previously (14, 15). HEK293 were maintained at 37°C in DMEM Dulbecco's modified Eagle's medium supplemented with 10% fetal bovine serum, 2 mM L-glutamine, 100 U/ml penicillin G, and 100 µg/ml streptomycin. For transient transfection, HEK293 cells were transfected using the calcium phosphate precipitation method (16).

### Immunoprecipitation, GST pull-down, SDS PAGE and immunoblotting

These procedures were done as reported before (14). Briefly, cells were lysed in lysis buffer: 20 mM Tris/HCl, pH 7.5, 150 mM NaCl, 5 mM EDTA containing 1% NP-40, 1 mM Na_3_VO_4_, 10 µg/ml aprotinin and leupeptin, and 1 mM phenylmethylsulphonyl fluoride. Lysates were clarified by centrifugation at 15,000 rpm for 10 min. The clarified lysates were preabsorbed on protein G-Sepharose (GE Healthcare) and then incubated with antibody for 2 h, followed by overnight incubation with protein G-Sepharose beads. Immune complexes were washed three times in lysis buffer and resuspended in SDS sample buffer. Proteins resolved by SDS-PAGE were transferred to nitrocellulose membrane, which were immunoblotted with optimal dilutions of specific Abs followed by the appropriate anti-IgG-peroxidase-conjugate. Blots were developed by the enhanced chemiluminescence technique (ECL kit, GE Healthcare) according to the manufacturer's instructions. Pull-down of GST fusion proteins was done with Glutathione-Sepharose beads (GE Healthcare) incubated with the clarified lysates for 2 hr. Then the complexes were washed and processed as explained above for the immunoprecipitation.

### In vitro dephosphorylation assay

HEK293T cells were transfected with the indicated plasmids to produce the proteins used as substrates and cells were treated with pervanadate to induce tyrosine phosphorylation of proteins. Cells were lysed in lysis buffer and the clarified lysates were immunoprecipitated with the appropriate antibodies. Washed immunocomplexes were incubated with 1 µg of YopH or YopH D356A at 4°C during the indicated times. Dephosphorylation of proteins in the immunocomplexes was detected by Western blot using the 4G10 antibody.

**Luciferase Assays—**Transfection of Jurkat T cells and assays for luciferase activity were performed as described previously (28–30). Briefly, 20×10^6^ Jurkat cells were transfected with 10 µg empty pEF5HA vector alone or YopH plasmids, along with 2 µg of NFAT/AP-1-luc (or other reporters) and 1 µg of a *Renilla* luciferase reporter for normalization. Cells were stimulated with anti-TCR plus anti-CD28 antibodies 24 hr after transfection for the last 6 hr. Cells were lysed then and processed to measure the luciferase activity with the Dual Luciferase system (Promega) according to the manufacturer's instructions.

**Statistics—**For statistical analysis of data, unpaired Student's t test was performed (PRISM version 4.0; GraphPad) as appropriate. Values of p<0.05 were considered significant.

## References

[1] Titball RW, Leary SE (1998). Plague.. Br Med Bull.

[2] Hinnebusch BJ (1997). Bubonic plague: a molecular genetic case history of the emergence of an infectious disease.. J Mol Med.

[3] Christie AB (1982). Plague: review of ecology.. Ecol Dis.

[4] Cleri DJ, Vernaleo JR, Lombardi LJ, Rabbat MS, Mathew A (1997). Plague pneumonia disease caused by Yersinia pestis.. Semin Respir Infect.

[5] Titball RW, Williamson ED (2001). Vaccination against bubonic and pneumonic plague.. Vaccine.

[6] Friedlander AM, Welkos SL, Worsham PL, Andrews GP, Heath DG (1995). Relationship between virulence and immunity as revealed in recent studies of the F1 capsule of Yersinia pestis.. Clin Infect Dis.

[7] Cornelis GR, Boland A, Boyd AP, Geuijen C, Iriarte M (1998). The virulence plasmid of Yersinia, an antihost genome.. Microbiol Mol Biol Rev.

[8] Cornelis GR, Wolf-Watz H (1997). The Yersinia Yop virulon: a bacterial system for subverting eukaryotic cells.. Mol Microbiol.

[9] Cornelis GR (2000). Molecular and cell biology aspects of plague.. Proc Natl Acad Sci U S A.

[10] Cheng LW, Schneewind O (2000). Yersinia enterocolitica TyeA, an intracellular regulator of the type III machinery, is required for specific targeting of YopE, YopH, YopM, and YopN into the cytosol of eukaryotic cells.. J Bacteriol.

[11] Persson C, Nordfelth R, Holmstrom A, Hakansson S, Rosqvist R (1995). Cell-surface-bound Yersinia translocate the protein tyrosine phosphatase YopH by a polarized mechanism into the target cell.. Mol Microbiol.

[12] Guan KL, Dixon JE (1990). Protein tyrosine phosphatase activity of an essential virulence determinant in Yersinia.. Science.

[13] Bliska JB, Guan KL, Dixon JE, Falkow S (1991). Tyrosine phosphate hydrolysis of host proteins by an essential Yersinia virulence determinant.. Proc Natl Acad Sci U S A.

[14] Stuckey JA, Schubert HL, Fauman EB, Zhang ZY, Dixon JE (1994). Crystal structure of Yersinia protein tyrosine phosphatase at 2.5 A and the complex with tungstate.. Nature.

[15] Montagna LG, Ivanov MI, Bliska JB (2001). Identification of residues in the N-terminal domain of the Yersinia tyrosine phosphatase that are critical for substrate recognition.. J Biol Chem.

[16] Evdokimov AG, Tropea JE, Routzahn KM, Copeland TD, Waugh DS (2001). Structure of the N-terminal domain of Yersinia pestis YopH at 2.0 A resolution.. Acta Crystallogr D Biol Crystallogr.

[17] Black DS, Bliska JB (1997). Identification of p130Cas as a substrate of Yersinia YopH (Yop51), a bacterial protein tyrosine phosphatase that translocates into mammalian cells and targets focal adhesions.. Embo J.

[18] Persson C, Carballeira N, Wolf-Watz H, Fallman M (1997). The PTPase YopH inhibits uptake of Yersinia, tyrosine phosphorylation of p130Cas and FAK, and the associated accumulation of these proteins in peripheral focal adhesions.. Embo J.

[19] Andersson K, Carballeira N, Magnusson KE, Persson C, Stendahl O (1996). YopH of Yersinia pseudotuberculosis interrupts early phosphotyrosine signalling associated with phagocytosis.. Mol Microbiol.

[20] Ernst JD (2000). Bacterial inhibition of phagocytosis.. Cell Microbiol.

[21] Aepfelbacher M, Zumbihl R, Ruckdeschel K, Jacobi CA, Barz C (1999). The tranquilizing injection of Yersinia proteins: a pathogen's strategy to resist host defense.. Biol Chem.

[22] Green SP, Hartland EL, Robins-Browne RM, Phillips WA (1995). Role of YopH in the suppression of tyrosine phosphorylation and respiratory burst activity in murine macrophages infected with Yersinia enterocolitica.. J Leukoc Biol.

[23] Yao T, Mecsas J, Healy JI, Falkow S, Chien Y (1999). Suppression of T and B lymphocyte activation by a Yersinia pseudotuberculosis virulence factor, yopH.. J Exp Med.

[24] Hamid N, Gustavsson A, Andersson K, McGee K, Persson C (1999). YopH dephosphorylates Cas and Fyn-binding protein in macrophages.. Microb Pathog.

[25] Black DS, Marie-Cardine A, Schraven B, Bliska JB (2000). The Yersinia tyrosine phosphatase YopH targets a novel adhesion-regulated signalling complex in macrophages.. Cell Microbiol.

[26] Alonso A, Bottini N, Bruckner S, Rahmouni S, Williams S (2004). Lck dephosphorylation at Tyr-394 and inhibition of T cell antigen receptor signaling by Yersinia phosphatase YopH.. J Biol Chem.

[27] Gerke C, Falkow S, Chien YH (2005). The adaptor molecules LAT and SLP-76 are specifically targeted by Yersinia to inhibit T cell activation.. J Exp Med.

[28] Viboud GI, Bliska JB (2005). Yersinia outer proteins: role in modulation of host cell signaling responses and pathogenesis.. Annu Rev Microbiol.

[29] Bliska JB, Clemens JC, Dixon JE, Falkow S (1992). The Yersinia tyrosine phosphatase: specificity of a bacterial virulence determinant for phosphoproteins in the J774A.1 macrophage.. J Exp Med.

[30] Ivanov MI, Stuckey JA, Schubert HL, Saper MA, Bliska JB (2005). Two substrate-targeting sites in the Yersinia protein tyrosine phosphatase co-operate to promote bacterial virulence.. Mol Microbiol.

[31] Koyasu S (2003). The role of PI3K in immune cells.. Nat Immunol.

[32] Hazeki K, Nigorikawa K, Hazeki O (2007). Role of phosphoinositide 3-kinase in innate immunity.. Biol Pharm Bull.

[33] Sauvonnet N, Lambermont I, van der Bruggen P, Cornelis GR (2002). YopH prevents monocyte chemoattractant protein 1 expression in macrophages and T-cell proliferation through inactivation of the phosphatidylinositol 3-kinase pathway.. Mol Microbiol.

[34] Sarmay G, Angyal A, Kertesz A, Maus M, Medgyesi D (2006). The multiple function of Grb2 associated binder (Gab) adaptor/scaffolding protein in immune cell signaling.. Immunol Lett.

[35] Bustelo XR (2001). Vav proteins, adaptors and cell signaling.. Oncogene.

[36] Hornstein I, Alcover A, Katzav S (2004). Vav proteins, masters of the world of cytoskeleton organization.. Cell Signal.

[37] Jascur T, Gilman J, Mustelin T (1997). Involvement of phosphatidylinositol 3-kinase in NFAT activation in T cells.. J Biol Chem.

